# Efficacy and Safety of Lobaplatin-TACE in the Treatment of Primary Hepatocellular Carcinoma: A Retrospective Study

**DOI:** 10.2174/1871520622666220601115458

**Published:** 2023-01-27

**Authors:** Haohao Lu, Chuansheng Zheng, Bin Liang, Bin Xiong

**Affiliations:** 1 Department of Radiology, Union Hospital, Tongji Medical College, Huazhong University of Science and Technology, Jiefang Avenue #1277, Wuhan, 430022, China;; 2 Hubei Province Key Laboratory of Molecular Imaging, Wuhan, 430022, China

**Keywords:** Lobaplatin, lipiodol emulsion, TACE, Transarterial chemoembolization, hepatocellular carcinoma, tumor response, survival rate

## Abstract

**Purpose:**

To investigate the safety and efficacy of lobaplatin-TACE in treating primary hepatocellular carcinoma.

**Methods:**

The data of 536 patients who underwent TACE in the interventional department from January 2016 to January 2020 were collected. Patients were divided into two groups according to the chemotherapeutic drugs used in TACE.: the epirubicin-TACE group (N = 260) and the lobaplatin-TACE group (N = 276). Primary study endpoint: (1) The tumor response after TACE; (2) The survival rates; Secondary study endpoints:(1) Changes in liver function and blood routine before and after TACE; (2) Occurrence of the post-embolization syndrome and infection after TACE.

**Results:**

The ORR was 35.0% in the epirubicin-TACE group and 51.1% in the lobaplatin-TACE group (*p*=0.001). The DCR was 73.1% in the epirubicin-TACE group and 82.2% in the lobaplatin-TACE group (*p*=0.011). The 6-month, 9-month, 12-month, and 15-month survival rates were higher in the lobaplatin-TACE group than in the epirubicin-TACE group (*p*=0.029, *p*=0.001, *p*=0.005, *p*=0.002). mOS: Epirubicin-TACE group,14.8 months; Lobaplatin-TACE group,18.6 months (*p*=0.007). mPFS: Epirubicin-TACE group,9.5 months; Lobaplatin-TACE group,12.8 months (P =0.000). There was no statistical difference in ALT, AST, total bilirubin and Leucocyte after TACE between the two groups (*p*=0.343, *p*=0.368, *p*=0.288, *p*=0.359). The platelet decrease after TACE was more significant in the lobaplatin-TACE group than in the epirubicin-TACE group (*p*=0.046). There was no statistical difference in the incidence rate of abdominal pain, fever and infection after TACE between the two groups (*p*=0.502, *p*=0.602, *p*=0.726). The incidence of vomiting after TACE in the lobaplatin-TACE group was higher than that in the epirubicin-TACE group (*p*=0.003).

**Conclusion:**

Lobaplatin-TACE has a higher tumor response rate and survival rate. Lobaplatin-TACE is a safe and effective treatment strategy; it is worthy of clinical application.

## 1 INTRODUCTION

Primary hepatocellular carcinoma is a malignant tumor with high morbidity and mortality [1, 2, 3], and it seriously threatens people's lives. Because there are no obvious symptoms and signs in the early stage of the disease, most patients are found in the middle and advanced stages and lose the chance of surgery. Transarterial chemoembolization (TACE) has been performed since the late1970s [4, 5]. The European Association for the Study of the Liver (EASL) guidelines recommend TACE for unresectable, Child-Pugh A or B multiple HCC with no vascular invasion [BCLC-B stage (intermediate-stage) HCC]. TACE can significantly prolong the overall survival(OS) and progression-free survival(PFS) of patients and benefit more and more patients with hepatocellular carcinoma [6, 7]. TACE includes Conventional transarterial chemoembolization (C-TACE) and Drug-eluting beads transarterial chemoembolization (DEB-TACE). In both C-TACE and DEB-TACE, we need to use chemotherapeutic agents. The types of chemotherapeutic drugs used in TACE may affect the efficacy of patients. The most commonly used and classical chemotherapeutic agents in TACE are anthracycline antineoplastic agents, such as epirubicin. Platinum is also a commonly used drug for hepatocellular carcinoma chemotherapy [8, 9]. Lobaplatin is a third-generation anti-tumor drug with good water solubility, strong anti-tumor activity, no cross-resistance with other platinum, and low toxicity. This study aimed to investigate the safety and efficacy of using lobaplatin-lipiodol emulsion in TACE for primary hepatocellular carcinoma.

## 2 MATERIALS AND METHODS

### 2.1 General Information

The data of 536 patients who underwent TACE in the Department of Interventional Therapy, Union Hospital, Tongji Medical College, Huazhong University of Science and Technology were collected from January 2016 to January 2020. This is a retrospective study. Inclusion criteria:(1) Clinical or pathological diagnosis of primary hepatocellular carcinoma; (2) Child-Pugh classification of liver function [10] A or B, performance score (ECOG) 0-2 points; (3) Aged 18-70 years old; (4) No use of molecular targeted drugs or immunotherapy during treatment. Exclusion criteria: (1) Child-Pugh classification of liver function C, performance score (ECOG) > 2 points; (2) severe coagulation dysfunction and can not be corrected; (3) cachexia or distant tumor metastasis; (4) complete portal vein occlusion and collateral vessels; (5) renal insufficiency;(6) total bilirubin>51.3 umol/L. The patients were reexamined every three months, and TACE was performed as needed according to the reexamination results. The number of TACE treatments for patients was 2–5 times. The patients were divided into two groups according to the chemotherapeutic drugs used in TACE: the group using lipiodol + epirubicin emulsion for TACE (epirubicin-TACE group, N = 260) and the group using lipiodol + lobaplatin emulsion for TACE (lobaplatin-TACE group, N = 276). All patients had liver cirrhosis on imaging examination. The baseline data collected before TACE included gender, age, etiology of liver cirrhosis, WBC, platelet, ALT, AST, total bilirubin, preoperative Child-Pugh classification of liver function, BCLC staging and ECOG score. The chemotherapeutic drugs and the amount of lipiodol used in TACE were recorded.

### 2.2 Methods

After disinfection, draping, and local anesthesia of the puncture site with 2% lidocaine, the right femoral artery was punctured using the Seldinger technique and a 5F vascular sheath (TERUMO5F-10CM, Terumo, Japan) was placed. The feeding artery of the tumor was identified by catheterization with a 5F Yashino catheter (Terumo, Japan) to the celiac trunk and superior mesenteric artery for angiography. A 2.7 F microcatheter (Terumo, Japan) was then used to super selectively cannulate into the tumor feeding artery. Embolization was performed by slowly injecting an appropriate amount of iodized oil emulsion and supplementing embolization with 300-500 um gel foam particles (CFDA 20193131657, Hangzhou ALICON Pharmaceutical Technology Co., Ltd, China), and the embolization endpoint was a stagnation of forwarding blood flow in the tumor feeding artery. Chemotherapeutic drugs used during TACE are divided into two types: (1) lobaplatin 50 mg; (GYZZ H20050308, Hainan Chang'an International Pharmaceutical Co., Ltd., China) (2) epirubicin 30 mg. (GYZZ H19990280, Zhejiang Hisun Pharmaceutical Co., Ltd., China) The dose setting of these chemotherapeutic drugs was based on the results of previous studies in our center, which found that increasing the dosage of chemotherapeutic drugs in TACE did not increase the efficacy of TACE but increased the side effects. The amount of lipiodol (GYZZ H20163348, Jiangsu Hengrui Pharmaceutical Co., Ltd, China) was 5–20ml. Composition of epirubicin-TACE group lipiodol emulsion: lipiodol+epirubicin; Composition of lobaplatin-TACE group lipiodol emulsion: lipiodol+lobaplatin.

### 2.3 Outcome Measures

Primary study endpoints: (1) The efficacy evaluation of tumor response after TACE in the two groups [complete response (CR), partial response (PR), stable disease (SD), progressive disease (PD)]; (2) Objective response rate (ORR) and Disease control rate (DCR) after TACE in the two groups; (3) The survival rates at 3 months, 6 months, 9 months, 12 months and 15 months in the two groups;(4) Overall survival (OS), Progression-Free Survival (PFS).

The efficacy of tumor response after TACE was evaluated in both groups, using the imaging data before and after the last TACE for efficacy evaluation, and the mRECIST criteria were used as the evaluation criteria [11, 12].

Secondary study endpoints: (1) Changes in liver function (ALT, AST, total bilirubin) before and after TACE in the two groups; (2) Changes in blood routine examination (WBC and PLT count) before and after TACE in the two groups; (3) Incidence of post-TACE embolism syndrome (including abdominal pain, fever, vomiting) in the two groups; (4) Incidence of infection after TACE in the two groups;

The liver function changes and blood routine examination before and after TACE in the two groups were compared with the results before and after the first TACE. Blood routine and liver function were reexamined one week after TACE.

### 2.4 Statistical Methods

Statistical analysis was performed using SPSS software (Version 24.0, IBM, Armonk, New York). Enumeration data were described by the number of cases (expressed as a percentage), and the difference between the two groups was analyzed by the Chi-square test, including Pearson Chi-Square and Fisher's Exact Test. Measurement data were expressed as mean ± standard deviation, and differences between two groups were analyzed by two independent samples or paired sample t-test. *p*<0.05 was considered to indicate a statistically significant difference.

## 3 RESULTS

### 3.1 Basic Information

There were no statistical differences in the gender, Child-Pugh classification of liver function, etiology of liver cirrhosis, tumor BCLC stage and ECOG score before TACE between the Epirubicin-TACE group and Lobaplatin-TACE group (Chi-square test was used with *p*-value > 0.05, Table **1**). The average age was 46.9±12.3 years in the epirubicin-TACE group and 47.8±13.0 years in the lobaplatin-TACE group; The ALT was 42.8 ± 18.6 U/L in epirubicin-TACE group and 41.8 ± 19.2 U/L in the lobaplatin-TACE group; The AST was 40.4 ± 20.9 U/L in epirubicin-TACE group and 42.6 ± 18.9 U/L in the lobaplatin-TACE group; The total bilirubin was 17.6 ± 9.0 umol/L in epirubicin-TACE group and 18.4 ± 9.6 umol/L in the lobaplatin-TACE group; The white blood cells were 3.96 ± 1.24 G/L in epirubicin-TACE group and 3.86 ± 1.88 G/L in the lobaplatin-TACE group; The platelets was 111.7 ± 52.3G/L in epirubicin-TACE group and 110.3 ± 56.2G/L in the lobaplatin-TACE group. Comparisons between the two groups were performed using the t-test with P-value > 0.05 and no statistical difference. (Table **2**) There was no statistical difference in the dosage of lipiodol in TACE between the epirubicin-TACE group and the lobaplatin-TACE group. (Chi-square test was used with *p*-value > 0.05, Table **3**).

### 3.2 Occurrence of Post-embolization Syndrome and Infection after TACE

In the epirubicin-TACE group, 90 patients had abdominal pain, with an incidence of 34.6%; In the lobaplatin-TACE group, 88 patients had abdominal pain, with an incidence of 31.9% (*p*>0.05). In the epirubicin-TACE group, 111 patients(42.7%) had a fever; In the lobaplatin-TACE group, 124 patients(44.9%) had a fever (*p*>0.05). In the epirubicin-TACE group, 58 patients(22.3%) had vomiting; In the lobaplatin-TACE group, 93 patients(33.7%) had vomiting (*p*<0.05). Referring to Common Terminology Criteria for Adverse Events(CTCAE 5.0), the patient's abdominal pain, fever, and vomiting were all grade 1-2, and no grade 3-4 adverse events occurred. In the epirubicin-TACE group, 8 patients(3.1%) had an infection; In the lobaplatin-TACE group, 10 patients had an infection(3.6%) (*p*>0.05). The Chi-square test, including Pearson Chi-Square and Fisher's Exact Tests, were used to compare the two groups, and P values < 0.05 were statistically different (Table **4**).

### 3.3 Changes in Liver Function and Blood Routine Tests of Patients after TACE (Re-examination One Week after the First TACE)

The ALT was 50.2 ± 23.0 U/L in the epirubicin-TACE group and 52.0 ± 21.5 U/L in the lobaplatin-TACE group (*p*>0.05); The AST was 51.3 ± 19.7 U/L in the epirubicin-TACE group, and 52.9 ± 21.8 U/L in the lobaplatin-TACE group (*p*>0.05); The total bilirubin was 18.6 ± 8.9 umol/L in the epirubicin-TACE group, and 19.4 ± 8.5 umol/L in the lobaplatin-TACE group (*p*>0.05); The white blood cells were 4.76 ± 2.03G/L in epirubicin-TACE group and 4.58 ± 2.55 G/L in the lobaplatin-TACE group (*p*>0.05); The platelets was 86.2 ± 39.1 G/L in epirubicin-TACE group and 79.5 ± 38.5G/L in the lobaplatin-TACE group (*p*<0.05). Comparisons between the two groups were performed using the t-test, and *p* values < 0.05 were statistically different (Table **5**).

### 3.4 Efficacy Assessment of Tumor Response after TACE (Efficacy Evaluation was Performed Using the Image Data Before and After the Last TACE Two Radiologists did this According to mRECIST Criteria)

16 patients(6.2%) achieved CR in the epirubicin-TACE group, 27 patients (9.8%) achieved CR in the lobaplatin-TACE group; 75 patients (28.8%) achieved PR in the epirubicin-TACE group, 114 patients (41.3%) achieved PR in the lobaplatin-TACE group; 99 patients (38.1%) achieved SD in the epirubicin-TACE group, 86 patients (31.2%) achieved SD in the lobaplatin-TACE group; 70 patients (26.9%) achieved PD in the epirubicin-TACE group, 49 patients (17.8%) achieved PD in the lobaplatin-TACE group (*p*<0.05). The ORR was 91 (35.0%) in the epirubicin-TACE group, and the ORR was 141 (51.1%) in the lobaplatin-TACE group. The DCR was 190 (73.1%) in the epirubicin-TACE group, and the DCR was 227 (82.2%) in the lobaplatin-TACE group. The Chi-square test, including Pearson Chi-Square and Fisher's Exact Test, was used to compare the two groups, and P values < 0.05 were statistically different (Table **6**).

### 3.5 Patient Survival Rate

Epirubicin-TACE group *vs.* lobaplatin-TACE group: 239 (91.9%) *vs.* 261 (94.6%) at 3 months (*p*>0.05); 198 (76.2%) *vs.* 231 (83.7%) at 6 months (*p*<0.05); 164 (63.1%) *vs.* 210 (76.1%) at 9 months (*p*<0.05); 135 (51.9%) *vs.* 176 (63.8%) at 12 months (*p*<0.05); 105 (40.4%) *vs.* 149 (54.0%) at 15 months (*p*<0.05); Chi-square test was used for comparison between the two groups, including Pearson Chi-Square and Fisher's Exact Test, with statistical difference at *p*-value < 0.05 (Table **7**).

### 3.6 OS and PFS in the Two Groups

mOS: Epirubicin-TACE group,14.8 months (95% CI 12.9-16.8 months); Lobaplatin-TACE group,18.6 months (95% CI 16.1-21.1 months). As shown in Fig. (**1**), the Log-Rank test was used between the two groups. Lobaplatin-TACE was superior to Epirubicin-TACE (*p* =0.007).

mPFS: Epirubicin-TACE group,9.5 months (95% CI 8.9-10.2 months); Lobaplatin-TACE group,12.8 months (95% CI 12.1-13.5 months). As shown in Fig. (**2**), the Log-Rank test was used between the two groups. Lobaplatin-TACE was superior to Epirubicin-TACE (*p*=0.000).

### 3.7 Comparison of Liver Function and Blood Routine Examinations Before and After TACE Between Epirubicin-TACE Group and Lobaplatin-TACE Group (Compare the Results Before and After the First TACE)

In the epirubicin-TACE group: The ALT was 42.8 ± 18.6 U/L before TACE and 50.2 ± 23.0 U/L after TACE (*p*>0.05); The AST was 40.4 ± 21.0 U/L before TACE and 51.3 ± 19.7 U/L after TACE (*p*<0.05); The total bilirubin was 17.6 ± 9.0 umol/L before TACE and 18.6 ± 8.9 umol/L after TACE (*p*<0.05); The white blood cells were 3.96 ± 1.24 G/L before TACE and 4.75 ± 2.03 G/L after TACE (*p*<0.05); The platelets were 111.7 ± 52.3 G/L before TACE and 86.2 ± 39.1 G/L after TACE (*p*<0.05). In the lobaplatin-TACE group: The ALT was 41.8 ± 19.3 U/L before TACE and 52.0 ± 21.5 after TACE (*p*>0.05); The AST was 42.6 ± 18.9 U/L before TACE and 52.9 ± 21.8 U/L after TACE (*p*<0.05); The total bilirubin was 18.4 ± 9.6 umol/L before TACE and 19.4 ± 8.5 umol/L after TACE (*p*<0.05); The white blood cells were 3.86 ± 1.88 G/L before TACE and 4.58 ± 2.55 G/L after TACE (*p*<0.05); The platelets were 110.3 ± 56.2 G/L before TACE and 79.5 ± 38.5 G/L after TACE (*p*<0.05) (Table **8**).

A paired sample t-test was used, and a *p*-value < 0.05 indicated a statistical difference.

## 4 DISCUSSION

TACE is one of the mainstays of treatment for hepatocellular carcinoma.TACE was shown to improve median survival from 16 to 20 months. Its main principle is to intubate the catheter into the feeding artery of the tumor super selectively after establishing vascular access through femoral artery puncture and injecting chemotherapeutic drugs and embolic agents [13]. On the one hand, chemotherapeutic drugs induce apoptosis and inhibit tumor cell proliferation; on the other hand, after tumor supply artery embolization, it leads to tumor cell ischemia, hypoxia and necrosis.TACE is effective in treating HCC and plays a very important role in the treatment of HCC. It is one of the main means for the treatment of advanced HCC. There is no uniform standard for the chemotherapeutic agents used in TACE, and there are various chemotherapeutic agents used in TACE in clinical practice [14]. The most commonly used chemotherapeutic agents are anthracycline antineoplastic agents, such as epirubicin, which are even regimens in the control group of many clinical studies. Different chemotherapeutic agents in TACE may affect the efficacy after TACE [15]. As seen from chemotherapy regimens for hepatocellular carcinoma, platinum is also one of the recommended chemotherapeutic agents [16]. Lobaplatin is a third-generation platinum anti-tumor drug [17], which can hinder the replication and transcription of DNA by generating platinum-GG and platinum-AG intra-chain cross-links, thereby interfering with the tumor cell cycle and is characterized by good water solubility, wide anti-tumor spectrum, strong anti-tumor activity, no cross-resistance with other platinum and low toxic side effects. Lobaplatin is mainly used for the treatment of breast cancer [18], small cell lung cancer [19] and chronic myelogenous leukemia, and it has also been confirmed that lobaplatin also has a good effect on nasopharyngeal carcinoma [20], colorectal cancer [21, 22], gastric cancer [23], and hepatocellular carcinoma [24, 25]. It has also been reported in the literature of different centers that lobaplatin has also achieved good efficacy in TACE for hepatocellular carcinoma [26]. Chen *et al*. showed [27] that lobaplatin, the 3rd generation of anti-tumor platinum-based drugs, is less toxic and more effective than cisplatin in TACE. Sheng Peng *et al*. reported [28] that Lobaplatin-TACE combined with 125I seed implantation is favorable and safe for treating primary HCC.

In this study, we found a statistically significant difference in the response rate between the lobaplatin-TACE group and the epirubicin-TACE group. The ORR and DCR in the lobaplatin-TACE group were significantly higher than those in the epirubicin-TACE group (ORR: 51.1% *vs.* 35.0%; DCR: 82.2% *vs.* 73.1%), which may be related to the strong anti-tumor activity of lobaplatin. Finding the literature, there are reports on the combination of lobaplatin and various other chemotherapeutic agents in TACE, but there are few reports on using lobaplatin alone in TACE. A study by He *et al*. reported [29] that Triple-drug(50 mg epirubicin, 50 mg lobaplatin, 6 mg mitomycin C) TACE seems to benefit patients with HCC larger than 10 cm in particular compared with single-drug(50 mg epirubicin) TACE. This study found that the survival rate at 6 months, 9 months, 12 months and 15 months of the lobaplatin-TACE group was superior to that of the epirubicin-TACE group, with a statistical difference, which was also related to the strong anti-tumor effect, low toxicity and high safety of lobaplatin. TACE has been reported to affect patients' liver function in a short time due to hepatic artery embolization and simultaneous use of chemotherapeutic drugs. Wang *et al*. found [30] that lobaplatin has better efficiency in the aspects of patient's mean survival time and therapeutic response in TACE. This is consistent with the results of this study. A study by Zhou *et al*. reported [31] that Lobaplatin-based chemoembolization may elicit effective tumor response for recurrent HCCs and improve the overall survival of patients with unresectable HCC recurrence following orthotopic liver transplantation.

In this study, there was a statistically significant increase in postoperative AST and total bilirubin in both the lobaplatin-TACE group and the epirubicin-TACE group, which was consistent with the results of other studies. However, this study found that there was no significant difference in postoperative ALT, AST, and total bilirubin between the lobaplatin-TACE group and the epirubicin-TACE group, indicating that lobaplatin-TACE was as safe as the standard regimen of C-TACE (lipiodol+epirubicin) and did not cause uncontrollable damage to the patient's liver function. The most common side effects after TACE are post-embolization syndrome [32], including abdominal pain, fever, nausea and vomiting. More and more attention has been paid to post-embolization syndrome after TACE by doctors and patients [33]. According to the existing studies on the efficacy and safety of TACE, the incidence rate of the post-embolization syndrome after TACE was about 47.7% [7]. This study found that the incidence of postoperative fever was 44.9% *vs.* 42.7%, and postoperative abdominal pain was 31.9% *vs.* 34.6% in the lobaplatin-TACE group versus the epirubicin-TACE group, with no statistically significant difference. However, the incidence of postoperative vomiting was 33.7% *vs.* 22.3%, a statistically significant difference. The incidence of postoperative vomiting was higher in the lobaplatin-TACE group, which may be related to the higher emetogenic risk of platinum than epirubicin. Still, with the advent of new antiemetics and the update of antiemetic regimens, the occurrence of postoperative vomiting in TACE patients can be effectively reduced by using drugs. Zhao *et al*. showed [34] that Lobaplatin-based TACE is an effective and safe treatment for primary liver cancer, adverse reactions (III-IV grade) were not common, with only 4 cases of vomiting and 2 cases of thrombocytopenia (III grade). A rare side effect after TACE is an infection, which will seriously affect the recovery of patients once it occurs [35]. This study's data showed no significant statistical difference in the incidence of postoperative infection between the lobaplatin-TACE group and the epirubicin-TACE group (3.6% *vs.* 3.1%). A study by Muhammet Arslan *et al*. reported [36] that the formation of liver abscesses after TACE is a rare but serious complication. In their research, liver abscesses were formed after treatment in four of the 163 (2.4%) patients and four (1.3%) of the 313 chemoembolization procedures. It is similar to the incidence in this study. The most common adverse effects of chemotherapeutic drugs are liver function damage, gastrointestinal reactions, and hematologic toxicity. Platinum chemotherapeutic agents are prone to myelosuppression [37]. A study by Wu *et al*. reported [38] that the main side effects of lobaplatin were myelosuppression. Twenty-five patients (21.9%) had grade 3/4 neutrophil suppression, and 18 patients (15.8%) had grade 3/4 thrombocytopenia. The results of this study showed that platelets after TACE in the lobaplatin-TACE group and epirubicin-TACE group were significantly reduced compared with those before TACE (110.3 ± 56.2 *vs.* 79.5 ± 38.5; 111.7 ± 52.3 *vs.* 86.2 ± 39.1), with a statistically significant difference. Meanwhile, the postoperative platelet decrease was more significant in the lobaplatin-TACE group than in the epirubicin-TACE group (79.5 ± 38.5 *vs.* 86.2 ± 39.1), with a statistically significant difference, which may be consistent with the fact that platinum-based chemotherapy drugs are prone to adverse reactions of myelosuppression, which is consistent with other results, but the postoperative platelet value was > 50.0 G/L in the lobaplatin-TACE group, which is in a relatively safe range. Lv *et al*. reported [39] that for HCC patients with normal pre-intervention platelet levels, the incidences of mild decrease, moderate decrease and severe decrease after intervention were 16.50%, 10.47% and 4.88%, respectively, and the incidences of long-term platelet reduction after intervention were 13.25%, 4.73% and 1.65% respectively. The long-term incidence of thrombocytopenia after interventional therapy was not high in TACE patients with HCC treated with lobaplatin alone, which was relatively safe. Thrombocytopenia due to lobaplatin has also been reported in treating other tumors. Lv *et al*. [40] reported that patients with advanced lung cancer developed thrombocytopenia using lobaplatin. Leukocytes increased significantly after TACE in both the lobaplatin-TACE group and the epirubicin-TACE group (3.86 ± 1.88 *vs.* 4.58 ± 2.55; 3.96 ± 1.24 *vs.* 4.75 ± 2.03), with a statistical difference. However, there was no statistically significant difference in leukocytes after TACE between the lobaplatin-TACE group and the epirubicin-TACE group (4.58 ± 2.55 *vs.* 4.75 ± 2.03). Patients in both groups had postoperative leukocytosis, which correlated with tumor necrosis absorption and aseptic inflammation after TACE [41, 42].

## CONCLUSION

There are various chemotherapeutic drugs used in TACE in clinical practice, and the choice of different chemotherapeutic drugs will affect the efficacy of TACE. Lobaplatin-TACE has a higher tumor response rate and survival rate. Lobaplatin-TACE is safe and has controllable postoperative side effects. Lobaplatin-TACE is a good therapeutic strategy and is worthy of clinical application.

The shortcomings of this study are that the sample size is limited, and it is a retrospective study. A multicenter, large-sample, prospective study can be conducted later to provide more help for clinical work.

## Figures and Tables

**Fig. (1):**
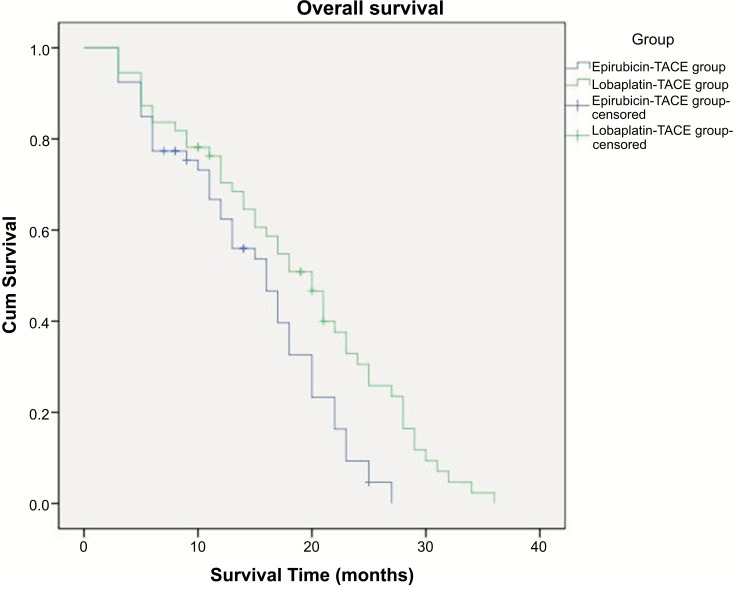
Overall survival of patients in two groups. mOS: Epirubicin-TACE group,14.8 months (95% CI 12.9-16.8 months); Lobaplatin-TACE group,18.6 months (95% CI 16.1-21.1 months).

**Fig. (2):**
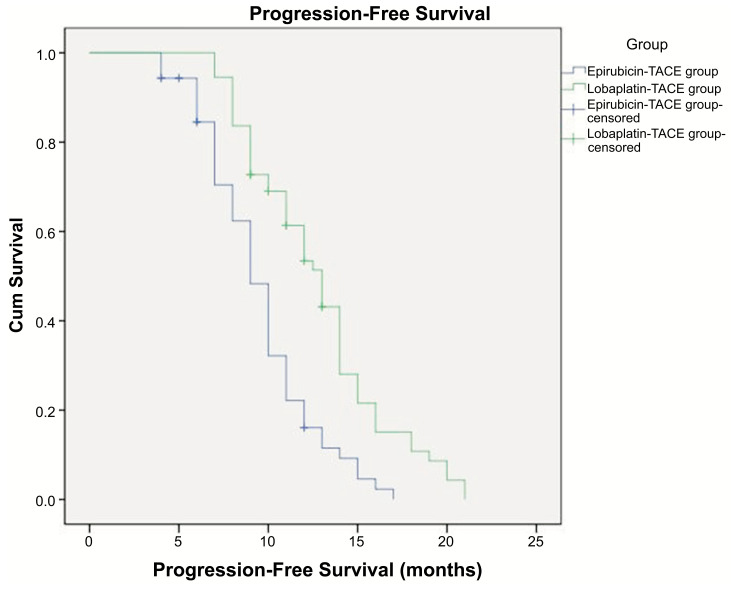
Progression-free survival time in the two groups. mPFS: Epirubicin-TACE group,9.5 months (95% CI 8.9-10.2 months); Lobaplatin-TACE group,12.8 months (95% CI 12.1-13.5 months).

**Table 1:** General information of the patients.

**-**			**Group**		**Chi-square test (*p*-value)**	
**-**	**-**	**-**	**Epirubicin-TACE group(N=260)**	**Lobaplatin-TACE group(N=276)**	**Pearson Chi-Square**	**Fisher's Exact Test**
Gender	Female	Count(%)	103(39.6%)	97(35.1%)	0.285	0.326
	Male	Count(%)	157(60.4%)	179(64.9%)		
Child-Pugh classification of liver function	A	Count(%)	185(71.2%)	195(70.7%)	0.898	0.924
	B	Count(%)	75(28.8%)	81(29.3%)		
Etiology of cirrhosis	Hepatitis B	Count(%)	221(85.0%)	228(82.6%)	0.453	0.483
	Hepatitis C	Count(%)	39(15.0%)	48(17.4%)		
BCLC staging	A	Count(%)	32(12.3%)	40(14.5%)	0.563	-
	B	Count(%)	148(56.9%)	145(52.5%)		
	C	Count(%)	80(30.8%)	91(33.0%)		
ECOG score	0	Count(%)	199(76.5%)	213(77.2%)	0.708	-
	1	Count(%)	42(16.2%)	39(14.1%)		
	2	Count(%)	19(7.3%)	24(8.7%)		

**Table 2:** Patient's age, liver function and Blood routine before TACE.

**-**	**Group**	**Mean**	**Std. Deviation**	**Levene's Test for Equality of Variances (*p*-value)**	**t-test (*p*-value)**
Age	Epirubicin-TACE group	46.9	12.3	0.138	0.435
	Lobaplatin-TACE group	47.8	13.0		
Preoperative ALT	Epirubicin-TACE group	42.8	18.6	0.142	0.546
	Lobaplatin-TACE group	41.8	19.2		
Preoperative AST	Epirubicin-TACE group	40.4	20.9	0.155	0.203
	Lobaplatin-TACE group	42.6	18.9		
Preoperative Bilirubin	Epirubicin-TACE group	17.6	9.0	0.115	0.335
	Lobaplatin-TACE group	18.4	9.6		
Preoperative leukocyte count	Epirubicin-TACE group	3.96	1.24	0.219	0.471
	Lobaplatin-TACE group	3.86	1.88		
Preoperative platelet count	Epirubicin-TACE group	111.7	52.3	0.350	0.767
	Lobaplatin-TACE group	110.3	56.2		

**Table 3:** Volume of Lipiodol used in TACE.

**-**			**Group**		**Chi-square test (*p*-value)**	
**-**	**-**	**-**	**Epirubicin-TACE group(N=260)**	**Lobaplatin-TACE group(N=276)**	**Pearson** **Chi-Square**	**Fisher's Exact Test**
Intraoperative lipiodol dose	<5ml	Count(%)	77(29.6%)	87(31.5%)	0.887	-
	5-10ml	Count(%)	120(46.2%)	123(44.6%)		
	>10ml	Count(%)	63(24.2%)	66(23.9%)		

**Table 4:** Incidence of post-embolization syndrome and Infection after TACE.

**-**			**Group**		**Chi-square test (*p*-value)**	
**-**	**-**	**-**	**Epirubicin-TACE group(N=260)**	**Lobaplatin-TACE group(N=276)**	**Pearson ** **Chi-Square**	**Fisher's Exact Test**
Postoperative fever	no	Count(%)	149(57.3%)	152(55.1%)	0.602	0.663
	yes	Count(%)	111(42.7%)	124(44.9%)		
Postoperative vomiting	no	Count(%)	202(77.7%)	183(66.3%)	0.003	0.004
	yes	Count(%)	58(22.3%)	93(33.7%)		
Postoperative abdominal pain	no	Count(%)	170(65.4%)	188(68.1%)	0.502	0.522
	yes	Count(%)	90(34.6%)	88(31.9%)		
Postoperative Infection	no	Count(%)	252(96.9%)	266(96.4%)	0.726	0.813
	yes	Count(%)	8(3.1%)	10(3.6%)		

**Table 5:** Patient's liver function and Blood routine after TACE.

**-**	**Group**	**Mean**	**Std. Deviation**	**Levene's Test for Equality of Variances(*p*-value)**	**t-test(*p*-value)**
Postoperative ALT	Epirubicin-TACE group	50.2	23.0	0.603	0.343
	Lobaplatin-TACE group	52.0	21.5		
Postoperative AST	Epirubicin-TACE group	51.3	19.7	0.122	0.368
	Lobaplatin-TACE group	52.9	21.8		
Postoperative Bilirubin	Epirubicin-TACE group	18.6	8.9	0.156	0.288
	Lobaplatin-TACE group	19.4	8.5		
Postoperative leukocyte count	Epirubicin-TACE group	4.76	2.03	0.304	0.359
	Lobaplatin-TACE group	4.58	2.55		
Postoperative platelet count	Epirubicin-TACE group	86.2	39.1	0.330	0.046
	Lobaplatin-TACE group	79.5	38.5		

**Table 6:** Efficacy assessments for tumor response after TACE.

**-**			**Group**		**Chi-square test (*p*-value)**	
**-**	**-**	**-**	**Epirubicin-TACE Group(N=260)**	**Lobaplatin-TACE Group(N=276)**	**Pearson ** **Chi-Square**	**Fisher's Exact Test**
Response evaluation	CR	Count(%)	16(6.2%)	27(9.8%)	0.002	-
	PR	Count(%)	75(28.8%)	114(41.3%)		
	SD	Count(%)	99(38.1%)	86(31.2%)		
	PD	Count(%)	70(26.9%)	49(17.8%)		
	ORR	Count(%)	91(35.0%)	141(51.1%)	0.001	0.003
	DCR	Count(%)	190(73.1%)	227(82.2%)	0.011	0.012

**Table 7:** Survival rate of patients at each stage after TACE.

**-**			**Group**		**Chi-square test (*p*-value)**	
**-**	**Death**	**-**	**Epirubicin-TACE Group(N=260)**	**Lobaplatin-TACE Group(N=276)**	**Pearson Chi-Square**	**Fisher's Exact Test**
3 months	no	Count(%)	239(91.9%)	261(94.6%)	0.222	0.232
	yes	Count(%)	21(8.1%)	15(5.4%)		
6 months	no	Count(%)	198(76.2%)	231(83.7%)	0.029	0.031
	yes	Count(%)	62(23.8%)	45(16.3%)		
9 months	no	Count(%)	164(63.1%)	210(76.1%)	0.001	0.001
	yes	Count(%)	96(36.9%)	66(23.9%)		
12 months	no	Count(%)	135(51.9%)	176(63.8%)	0.005	0.007
	yes	Count(%)	125(48.1%)	100(36.2%)		
15 months	no	Count(%)	105(40.4%)	149(54.0%)	0.002	0.002
	yes	Count(%)	155(59.6%)	127(46.0%)		

**Table 8:** Comparison of liver function and blood routine before and after TACE.

**-**	**-**	**Epirubicin-TACE Group**			**Lobaplatin-TACE Group**		
**-**	**-**	**Mean**	**Std. Deviation**	**Paired Samples Test(*p*-value)**	**Mean**	**Std. Deviation**	**Paired Samples Test(*p*-value)**
Pair 1	Preoperative ALT	42.8	18.6	0.157	41.8	19.3	0.114
	Postoperative ALT	50.2	23.0		52.0	21.5	
Pair 2	Preoperative AST	40.4	21.0	0.021	42.6	18.9	0.026
	Postoperative AST	51.3	19.7		52.9	21.8	
Pair 3	Preoperative Bilirubin	17.6	9.0	0.035	18.4	9.6	0.037
	Postoperative Bilirubin	18.6	8.9		19.4	8.5	
Pair 4	Preoperative leukocyte count	3.96	1.24	0.019	3.86	1.88	0.023
	Postoperative leukocyte count	4.75	2.03		4.58	2.55	
Pair 5	Preoperative platelet count	111.7	52.3	0.004	110.3	56.2	0.001
	Postoperative platelet count	86.2	39.1		79.5	38.5	

## Data Availability

Not applicable.
